# Rituximab and bilateral HSV epithelial keratitis in a patient with mucous membrane pemphigoid

**DOI:** 10.1186/s12348-018-0153-8

**Published:** 2018-08-23

**Authors:** W. Bernauer, S. Schuler, L. Borradori

**Affiliations:** 1 ÓMMA Eye Clinic, Theaterstrasse 2, CH-8001 Zurich, Switzerland; 2 University of Zürich, Zurich, Switzerland; 3 Department of Dermatology, University Hospital of Bern, Bern, Switzerland; 4 University of Bern, Bern, Switzerland

**Keywords:** Epithelial keratitis, Herpes simplex virus, Mucous membrane pemphigoid, Ocular inflammation, Rituximab

## Abstract

**Background:**

B cell depleting therapy is widely used for lymphoproliferative diseases and immune-mediated disorders, including mucous membrane pemphigoid. The latter is an autoimmune blistering disease affecting predominantly the mucosae potentially associated with devastating complications.

**Methods:**

A 71-year-old patient with severe mucous membrane pemphigoid involving ocular, oral pharyngeal and laryngeal involvement is described. To control the disease, the patient was given rituximab therapy in combination with oral corticosteroids. He subsequently experienced an epithelial herpes simplex virus keratitis in one eye and 3 months later in his fellow eye. Topical treatment with ganciclovir resulted in prompt recovery.

**Results:**

For the first time, a correlation between rituximab and bilateral epithelial herpes simplex virus keratitis is described.

**Conclusions:**

Although rituximab is a promising biologic agent for the treatment of autoimmune diseases, it bears the risk of reactivation of viral infections, including the onset of herpes simplex virus keratitis.

## Dear Editor

Rituximab is a B cell depleting anti-CD20 monoclonal antibody that is increasingly used to treat autoimmune disorders and B cell non-Hodgkin lymphoma. It has become an important therapeutic option for the treatment of refractory mucous membrane pemphigoid [1, 2]. As with other immunosuppressive agents, there is the risk of opportunistic infections or reactivations, including hepatitis B virus and herpes viruses (HSV) [3–5]. Here, we report a potential association between rituximab and de novo corneal infection with HSV.

## Clinical case

**Fig. 1:**
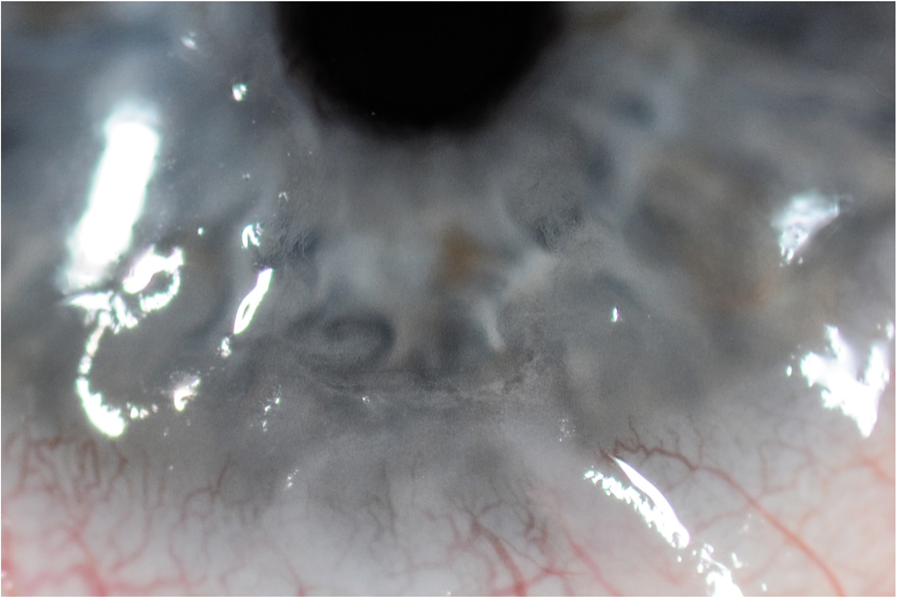
A new epithelial defect was seen at slit lamp examination

**Fig. 2:**
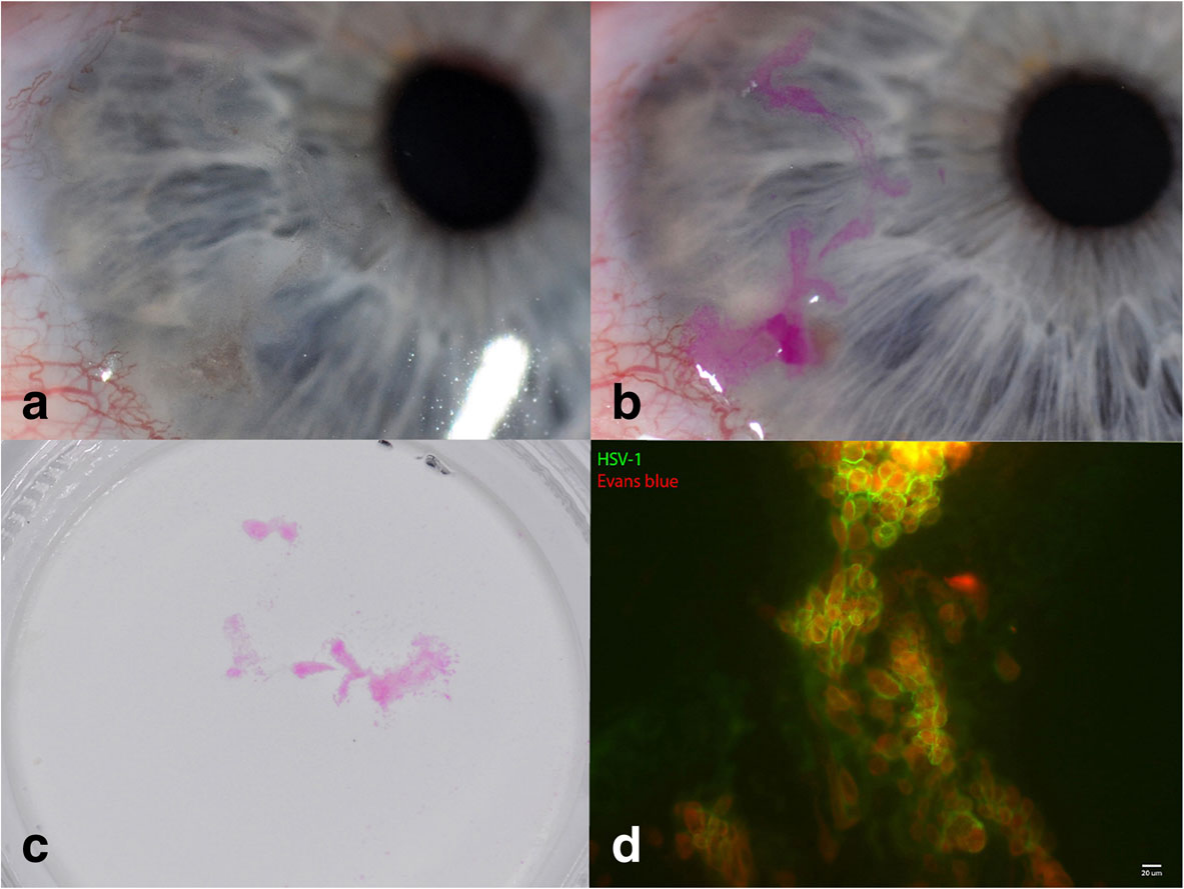
**a**–**d** A dense cataract and an epithelial keratitis

A 71-year-old male patient had a 3-year history of mucous membrane pemphigoid. He first presented with oral ulcerations, cicatrising conjunctivitis as well as cutaneous blisters. He was initially treated with prednisone 1 mg/kg/24 h and azathioprine 2 × 100 mg/24 h with subsequently progressive tapering of the oral corticosteroids. Azathioprine was stopped 18 months after the therapy start. Nevertheless, 2 years after initial diagnosis, he relapsed with severe oral and larynx involvement with dysphagia and respiratory distress. The latter required a tracheotomy with reintroduction of the immunosuppressive treatment. Prednisone 1 mg/kg/24 h and again azathioprine 2 × 50 mg/24 h were given over a period of 3 months. This was beneficial with regard to the skin lesions but had only little effect on the severe oral and laryngeal inflammation. Therefore, the patient received rituximab (MabThera), two times 1000 mg 2 weeks apart. Six weeks after the first infusion of rituximab, there was a significant regression of the oral lesions, but new ocular symptoms developed. The patient complained of foreign body sensation and of blurred vision in his right eye. Vision in this right pseudophacic eye had dropped to 0.5, and a new epithelial defect was seen at slit lamp examination (Fig. 1). The changes of the corneal epithelium were suggestive of a dendritic epithelial ulceration as seen in HSV keratitis. An impression cytology of the corneal surface was taken, which allowed to confirm the diagnosis of HSV epithelial keratitis. Topical treatment with ganciclovir gel 1.5 mg/g (Virgan) 4×/24 h was started. Seven days later, the corneal surface recovered completely and without any opacification. A maintenance therapy with ganciclovir gel 1.5 mg/g 1×/24 h was started. Noteworthy, to that date, there was no previous history of muco-cutaneous HSV disease. The immunosuppressive therapy was tapered to 40 mg prednisone/24 h and azathioprine 50 mg/24 h. Four months after the first infusion with rituximab, the patient developed new ocular symptoms in his left eye. He presented with an increasing foreign body sensation, vision 0.1 due a dense cataract and an epithelial keratitis (Fig. 2a, b). As for the right eye, HSV epithelial keratitis was confirmed. Treatment with ganciclovir 1.5 mg/g 4×/24 h resulted in a rapid response with resolution of the keratitis within 10 days. The patient was given a maintenance therapy with ganciclovir gel 1.5 mg/g 1×/24 h. There were no further episodes of HSV keratitis during the subsequent 12-month follow-up.

## Diagnostics and intervention

The clinical diagnosis of mucous membrane pemphigoid was confirmed based on the characteristic clinical features, consistent light microscopy findings and positive immunopathological findings. The direct immunofluorescence microscopy studies showed linear IgG and C3 deposits along the epidermal basement membrane. By indirect immunofluorescence microscopy using saline-split normal human skin as substrate, the patient had circulating autoantibodies reacting against the epidermal side of the split, while the ELISA-BP180 (MBL International, Japan) was positive (49.4 U/mL; normal value < 9 U/L). The lymphocyte count was reduced to 0.8 × 10^9^/L 6 months after the intensification of the immunosuppressive therapy, i.e. 2 months after the onset of HSV keratitis in the right eye.

For the diagnosis of the ocular surface changes, an impression cytology technique was used as described elsewhere (Fig. 2c) [6]. The impressions were processed, using monoclonal antibodies to assess the presence of HSV (fluorescein isothiocyanate-labelled mouse monoclonal antibodies to HSV-1 antigen, Evans Blue counterstain; MicroTrak HSV-1 Culture Identification/Typing Reagent, Trinity Biotech). The impressions were tested positive for HSV-1 in both eyes (Fig. 2d). Ganciclovir gel 1.5 mg/g was applied topically 4×/24 h to treat the superficial HSV keratitis for 10 days. Once the epithelial changes had resolved, a maintenance therapy with ganciclovir gel 1×/24 h was established. No topical steroids were applied.

## Discussion

This is the first report on bilateral epithelial HSV-1 keratitis in a patient with progressive severe mucous membrane pemphigoid. The keratitis first developed in one eye, and 3 months later in the fellow eye. HSV keratitis is usually regarded as a recurrence of a primary infection during childhood. In most cases, it presents as an unilateral condition [7, 8]. A compromised immune system, however, is a well-recognised risk factor for bilateral HSV keratitis [7, 8]. Therefore, patients with autoimmune disorders are at risk while on anti-inflammatory medication. Various immunosuppressive drugs have been linked to the reactivation of viral infections. They have also been reported for rituximab, including reactivations of polyomaviruses, hepatitis B virus and viruses of the herpes group (cytomegalovirus and Epstein-Barr virus) [3, 4]. However, there is no previous report on reactivation of HSV-1 keratitis or de novo corneal infection with HSV-1.

The sequence of therapy change in our patient strongly suggests a causal relation between rituximab use and the development of a first episode of HSV-1 keratitis in one eye, and later in the fellow eye. It is also conceivable that the previous immunosuppressive regimen with steroids and azathioprine has put our patient at additional risk for herpes infection.

Special clinical and laboratory monitoring of patients on immunosuppressive medications including rituximab, a highly effective biologic agent, is mandatory to exclude the development for severe infectious complications, such as in our patient.
